# Sex‐specific differences in the prevalence of intermediate hyperglycaemia states: A systematic review and meta‐analysis

**DOI:** 10.1111/dme.70293

**Published:** 2026-03-10

**Authors:** Louise Cooper, Ian Porter, Claire Meek, Simon J. Griffin, Amy Ahern

**Affiliations:** ^1^ Institute of Metabolic Science Epidemiology University of Cambridge Cambridge UK; ^2^ Health Services and Policy Research Group University of Exeter Medical School Exeter UK; ^3^ Leicester Diabetes Centre and Leicester NIHR Biomedical Research Centre University of Leicester, Leicester General Hospital Leicester UK; ^4^ Primary Care Unit, Department of Public Health and Primary Care University of Cambridge Cambridge UK

**Keywords:** diabetes, diagnosis, intermediate hyperglycaemia, screening

## Abstract

**Aims:**

To evaluate the sex‐specific prevalence of isolated impaired glucose tolerance, isolated impaired fasting glucose, combined impaired glucose tolerance and impaired fasting glucose and type 2 diabetes diagnosed by isolated glucose states.

**Methods:**

We searched MEDLINE, Cochrane Database of Systematic Reviews, Embase and CINAHL from inception to 22.04.25. Title and abstract screening, full‐text review, data extraction and risk of bias assessment (Hoy et al.) were conducted by two reviewers using pre‐defined eligibility criteria. Sex‐stratified prevalence and odds ratio for each intermediate hyperglycaemic state in women compared with men were pooled using a random‐effects meta‐analysis.

**Results:**

We identified 8 studies suitable for meta‐analysis, including 52,256 participants (25,263 women). The pooled prevalences (95% CI) of isolated impaired glucose tolerance, isolated impaired fasting glucose (ADA thresholds) and combined impaired glucose tolerance in women were respectively 8% (7%,9%), 15% (12%,18%) and 7% (5%, 9%) and for men 5% (4%,7%), 21% (12%, 32%) and 7% (5%, 10%). Compared with men, women had higher odds of having isolated impaired glucose tolerance: 1.42 (1.23, 1.65), 0.65 (0.44, 0.96) lower odds of isolated impaired fasting glucose: 0.65 (0.44, 0.96) and similar odds of combined impaired glucose tolerance and impaired fasting glucose: 0.85 (0.46, 1.57). There was moderate certainty evidence for pooled prevalence estimates and high certainty evidence for comparisons of iIGT and iIFG between sexes.

**Conclusions:**

There are sex‐specific disparities in the prevalence of different isolated intermediate hyperglycaemic states, which may contribute to underdiagnosis and undertreatment in primary care and introduce ascertainment bias into research and policy.

## Graphical abstract

There are sex‐specific disparities in the prevalence of different isolated intermediate hyperglycaemic states, which, in the context of clinical guidance to use FPG or HbA1c for identification, may contribute to underdiagnosis and undertreatment in primary care and introduce ascertainment bias into research and policy.

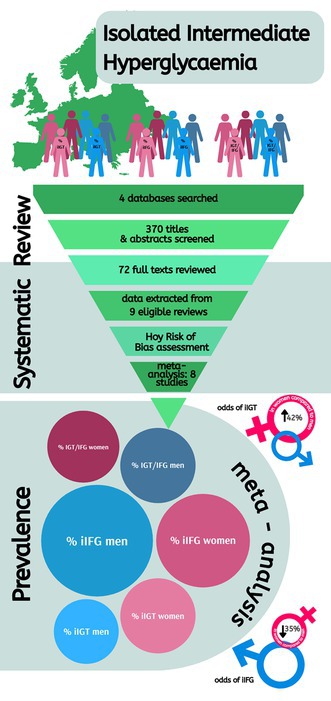

**What's new?**

- HbA_1c_ and fasting plasma glucose are neither specific nor sensitive for identifying isolated impaired glucose tolerance, and HbA_1c_ is neither specific nor sensitive for identifying isolated impaired fasting glucose.
- We estimate that the pooled odds of iIGT are 42% higher in women compared with men, and those of iIFG are estimated to be 35% lower.
- Policy and practice guiding the identification of IH may need to be reviewed to reduce the risk of underdiagnosis and undertreatment in clinical practice, as well as to address ascertainment bias in research and any sex‐based inequalities.

## 1 INTRODUCTION

Intermediate hyperglycaemia, also known as prediabetes,^1^ is a key risk factor for type 2 diabetes that presents in different states: impaired glucose tolerance (IGT), impaired fasting glucose (IFG) and combined IGT/IFG, Table 1. IGT is more common than IFG and affects more than 623 million adults globally, with 370 million adults expected to have IFG.^2^ Identification of people with IH is a key component of diabetes prevention programmes.

**TABLE 1:** Thresholds for prediabetes defined by different intermediate hyperglycaemic states.

Isolated IH state	Description
*Using WHO thresholds*	
iIGT	IGT 7.8–11.0 mmol/L with normal IFG <6.1 mmol/L
iIFG	IFG 6.1–6.9 mmol/L with normal IGT <7.8 mmol/L
T2D (isolated raised 2 h glucose)	2 h glucose ≥11.1 mmol/L with normal fasting glucose <6.1 mmol/L
T2D (isolated raised fasting glucose)	Fasting glucose ≥7.0 mmol/L with normal 2 h glucose <7.8
*Using ADA thresholds*	
iIGT	IGT 7.8–11.0 mmol/L with normal IFG <5.6 mmol/L
iIFG	IFG 5.6–6.9 mmol/L with normal IGT <7.8
T2D (isolated raised 2 h glucose)	2 h glucose ≥11.1 mmol/L with normal fasting glucose <5.6 mmol/L
T2D (isolated raised fasting glucose)	Fasting glucose ≥7.0 mmol/L with normal 2 h glucose <7.8

The pathophysiology of IFG and IGT differs, conferring different risks and patterns of progression to type 2 diabetes.^3^ IGT is associated with peripheral insulin resistance, resulting in poor glucose disposal 2 h after a 75 g glucose load (OGTT) and risk of macrovascular complications. IFG results from hepatic insulin resistance with reduced hepatic glucose clearance and is associated with microvascular complications.^3^ These two states occur independently as isolated IGT (iIGT) with normal fasting glucose and isolated IFG (iIFG) with normal glucose tolerance, or as combined IFG/IGT.^4^, ^5^ All three states can lead to hyperinsulinemia and type 2 diabetes,^4^, ^6^ the risk of type 2 diabetes is highest with combined IGT/IFG (relative risk compared with normoglycaemia 12.1^7^) followed by iIFG and iIGT.

Tests for glycaemia identify different IH subgroups and, therefore, different people. Though more costly and onerous for patients and practitioners, the oral glucose tolerance test (OGTT) will identify people with iIFG, iIGT, or combined IGT/IFG. The World Health Organisation (WHO) estimates that using fasting plasma glucose alone will miss 30% of cases.^8^ HbA_1c_ is easy to administer in primary care; however, in a meta‐analysis, Barry et al. (2017)^9^ demonstrated that HbA_1c_ is neither sensitive nor specific for identifying IH states. For example, it had a pooled sensitivity of 0.49 (95% CI: 0.40, 0.58) and specificity of 0.79 (95% CI: 0.73, 0.84) for identifying iIGT.^9^ This presents challenges in the development of policies that balance accurate identification with acceptability, usability and cost‐effectiveness.

As a consequence of these intersecting issues, many people with iIGT are likely to remain undiagnosed and undertreated. Sex differences in the prevalence of IGT and IFG have long been recognised^10^, ^11^ and were described in European populations by the Diabetes Epidemiology: Collaborative Analysis of Diagnostic Criteria in Europe (DECODE) study in 2003.^12^ Disparities in the prevalence of glucose states between the sexes may intersect with variable test sensitivity and specificity, resulting in unequal access to diagnosis and treatment. We aimed to assess sex‐specific prevalences of iIGT, iIFG and combined IGT/IFG in cross‐sectional and cohort studies of European people and conducted the first systematic review and meta‐analysis synthesising this evidence. We hypothesised that the prevalence of iIGT would be greater in women than in men and that of iIFG would be greater in men than in women.

## 2 METHODS

We registered this review with PROSPERO (CRD42023461180) and followed the JBIMES guidance on conducting systematic reviews of prevalence data, with additional guidance from the Cochrane Handbook of Systematic Reviews of Interventions.^13^, ^14^ We followed the Meta‐analysis of Observational Studies in Epidemiology reporting guidelines.^15^

### 2.1 Search terms

LC (PhD student) completed a PICOS concept map (Table 1, ESM 2) to identify key search terms. While the exposure: ‘oral glucose tolerance test’ and comparators: ‘fasting glucose test’, and HbA_1c_ are all MeSH terms, scoping searches revealed that they were not specific enough to yield relevant studies reporting true isolated IH states rather than those using WHO criteria for IH ascertainment. The WHO criterion for IGT (2 h plasma glucose 7.8–11.1 mmol/L) includes people with fasting plasma glucose that may also meet the criterion for IFG (<7.0 mmol/L). As such, keywords were used to identify any study yielding the outcomes: iIFG, iIGT and isolated HbA_1c_ (iHbA_1c_).

### 2.2 Search

LC searched MEDLINE (via OVID), Cochrane Database of Systematic Reviews, Embase (via OVID) and CINAHL from inception to 10th April 2025 with no limits or search restrictions. LC conducted forward and backward citation searches of included studies. Search strategies are listed in Table 2, ESM 2.

**TABLE 2:** Characteristics of included studies.

Authors	Study location	Cohort	Study aims	Period of data collection	Inclusion/exclusion criteria	Participation rate	Age range	Sex	IH state ascertainment Protocol/assay method
Cobb et al.^16^	Multiple	RISC	Use of plasma metabolites to distinguish IGT	2 years 2002–2004	Incl: ‘clinically healthy’	nr	30–60	m/f	Standardised fasting 75 g glucose OGTT Assay method nr ADA thresholds
Markus et al.^17^	Germany	KORA F4	Association between glucose/insulin markers and urinary albumin	2 years 2006–2008	Excl: known DM or <8 h fasting or missing OGTT+ values or other covariates	Baseline response rate 64.2% Follow up: 72.2%	32–82	m/f	Serum samples 75 g anhydrous glucose OGTT and hexokinase method ADA thresholds
Novoa et al.^18^	Spain	Telde	To assess the cardiovascular risk profile the degree of insulin resistance and beta cell secretion in a cohort of subjects with different categories of IGR IGT, IFG and combined IFG/IGT	nr	Known or newly diagnosed DM	1030/1193 = 86.3%	30–80	m/f	Standardised 75 g glucose OGTT Assay method nr ADA thresholds
Qiao et al.^19^	Finland	MONICA	Estimate risk of progression to diabetes	1 year 1987	None reported	81%	45–64	m/f	Standardised 75 g glucose OGTT (after >4 h fasting) Whole blood glucose determined with hexokinase glucose‐6‐phosphate dehydrogenate method. ADA thresholds
Satman et al.^20^	Turkey	TURDEP II IH	Estimate prevalence	6 months 2010	Incl: All residents	87%	≥20	m/f	Fasting capillary glucose 2 h capillary glucose after 75 g glucose Assay method nr ADA thresholds
Sparso et al.^21^	Denmark	Inter99	To refine the association signal within the 62‐Kb LD block that includes MTNR1B. This was done by direct genotyping of additional single‐nucleotide polymorphisms (SNPs) in LD (r2 0.70) with rs1387153 (including rs10830963) in large European populations	15.03.99–31.01.01	Birth years from 1939–1940 to 1969–1970‐ five‐year gaps at quintile birthday. Exclusion for alcoholism or drug abuse or linguistic problems	53.4% The participation rate was higher in younger women than in younger men, and it increased with increasing age until 55 years of age after which it declined.	NR	m/f	WHO standardised OGTT after ≥8 h fasting Assay method nr WHO thresholds
Tamayo et al.^22^	Germany	SHIP TREND	Estimate prevalence	4 years 2008–2012	Excl: known DM/<8 h fasting	50.1%	35–79	m/f	2 h post 75 g anhydrous glucose load Hexokinase method ADA thresholds
Tutuncu et al.^23^	Turkey	TURDEP II	Cut‐off points of hs‐CRP	6 months 2010	Excl: known DM/systemic disease with hs‐CRP≥	85%	≥20	m/f	Protocol nr HbA1c by turbidimetric inhibition immunoassay. Other assay methods nr ADA thresholds
Wang et al.^24^	Finland	METSIM	Concentration of lipoprotein particles in a population‐based study	5 years 2005–2010	Excl: prior DM	nr	45–70	m	2 h 75 g glucose OGTT Plasma glucose measured using enzymatic hexokinase photometric assay ADA thresholds

*Note*: American Diabetes Association (ADA) thresholds: IGT ≥7.8 and < 11.1 mmol/L with normal IFG <5.6 mmol/L IFG 5.6–6.9 mmol/L with normal IGT <7.8. World Health Organisation thresholds (WHO): IGT ≥7.8 and <11.1 mmol/L with normal IFG <6.1 mmol/L IFG 6.1–6.9 mmol/L with normal IGT <7.8 mmol/L.

### 2.3 Selection process

We uploaded the search to Covidence (RRID:SCR_016484) online systematic review management software.^25^ LC and IP independently screened title/abstract and full‐texts review using the eligibility criteria, with disagreements resolved by discussion. Eligible studies had randomly selected populations within the age range of 18–75 years, were conducted wholly or mainly in Europe and were cross‐sectional or cohort studies with baseline data reporting sex‐stratified numbers or prevalence of true isolated IGT (normal fasting glucose) and IFG (normal glucose tolerance) in European populations (using current WHO or ADA thresholds) to ascertain IH or type 2 diabetes (Table 1). A list of full‐text citations located and excluded, including the justification for exclusion, can be found in Table 3, ESM 2. No articles written in a language other than English were found. Abstracts were excluded if they lacked sufficient data for meta‐analysis or if no full text could be accessed. No unpublished studies were identified.

**TABLE 3:**
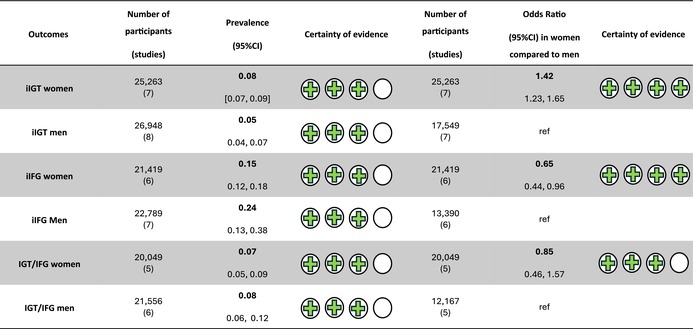
GRADE summary of evidence table.

### 2.4 Data collection

LC developed a data extraction tool and extracted the data, which was then checked by IP. Data specifying the sex‐specific prevalence of iIFG, iIGT, combined IGT/IFG and T2D diagnosed by isolated glucose states (Table 1) were extracted. For multiple reports of the same cohort, we only included the study with the most detailed information about study design, characteristics, outcomes and results, or the most participants included in the analysis.

We also extracted data on: author, publication date, type of study, name of cohort study, country where study was conducted, geographic area/boundary, study aims, recruitment method, participants age, population characteristics, sampling methods, assay era, OGTT protocol followed y/n (overnight fast and FPG test), followed venous plasma glucose after ingestion of 75 g oral glucose load (75 g), date or period of data collection, inclusion/exclusion criteria, participation rate, missing data, sex, criteria used to ascertain IH and T2D, prevalence data (with confidence intervals) or numerical data: total participants, number of women and men, sex stratified numbers with: iIGT, iIFG, iHbA_1c_, combined IGT/IFG, T2D diagnosed by isolated IH state, potential moderators/confounders: mean BMI and waist circumference for each subgroup.

### 2.5 Methodological quality assessment

LC assessed quality using the Hoy Risk of Bias tool^26^ and this was checked by IP. The Hoy Risk of Bias Tool was designed to evaluate the risk of bias in prevalence studies and was chosen instead of the Joanna Briggs tool specified in the study protocol because the majority of the criteria were identical and it provided additional structure for decision‐making: detailed examples of high risk and low risk of bias for each question and a points system with clear cut‐offs for low (8–10), moderate (5–7) and high risk of bias (0–4).

Publication bias was assessed using Harbord, Begg and Egger tests as there were less than 10 studies available for assessment by funnel plot.

### 2.6 Certainty of evidence assessment

LC and IP used the Grading of Recommendations Assessment, Development, and Evaluation (GRADE)^27^, ^28^ system to rate the certainty of evidence for the meta‐analysis.

### 2.7 Synthesis

Studies were eligible for statistical synthesis if they presented numbers of cases (numerator) and numbers of men and women (denominator) separately, or if it was possible to back‐calculate these data from the prevalence and denominator.

We conducted a meta‐analysis to generate pooled prevalence proportions (with 95% confidence intervals) for each IH state. We conducted a further meta‐analysis to generate the pooled Odds Ratio (with 95% confidence intervals) of each IH state occurring in women compared with men. Meta‐analyses are presented in forest plots.

We used the statistical software STATS Direct version 3^29^ to conduct the analyses. The proportions were transformed using the Freeman.

Tukey double arcsine transformation to stabilise the variance. Then random‐effects meta‐analysis was conducted using the DerSimonian and Laird method^30^ to account for ‘within‐study variability and that arising from differences between studies.^14^

We explored heterogeneity in three ways. We visually appraised the forest plots of the meta‐analyses, and we examined the *I* ^2^ statistic (with a 95% confidence interval) to evaluate the proportion of variance observed in the forest plot that is variance in the true prevalence, which is not due to chance.^31^

Whilst we anticipated in our protocol that our results would be stratified by diagnostic criteria, only one study reported data using the WHO thresholds. Therefore, we conducted subgroup analyses to assess the impact of including this study. In addition, two included studies were small, and the period of data collection across the included studies was long and historic (1987–2012). We therefore conducted sensitivity analyses to explore these two factors. These analyses are reported narratively.

## 3 RESULTS

### 3.1 Studies included in the meta‐analyses

**FIGURE 1:**
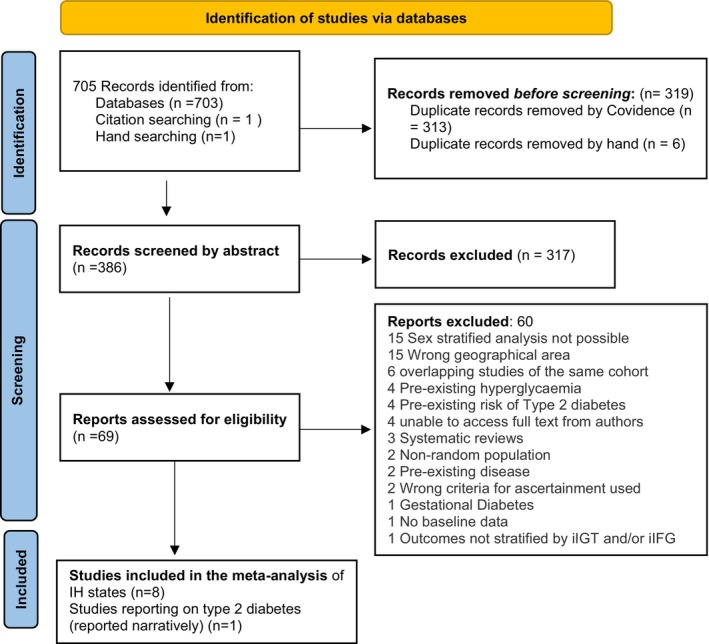
PRISMA flowchart.

We identified 705 references, and after de‐duplication, 386 references were screened for inclusion. Sixty‐nine references were read in full, and 9 met the inclusion criteria and were included in the review (Figure 1). Eight of these were included in the meta‐analysis (52,256 participants: 25,263 women, 26,993 men), and one is reported narratively. Notably, 15 studies met all the inclusion criteria but did not present sex‐stratified data and so could not be included in the meta‐analysis.

Four studies estimated the prevalence of IH and four explored associations of IH/type 2 diabetes with other diseases or markers. Most studies used the American Diabetes Association thresholds to identify iIFG (5.6–6.9 mmol/L with normal postprandial glucose levels (<7.8 mmol/L)), whereas Sparso^21^ used the WHO thresholds (6.1–6.9 mmol/L). The period of data collection ranged from 6 months to 5 years, though Wang^24^ and Cobb^16^ did not specify the data collection period clearly. Seven studies sampled people >30 years, two of which >45 years: Qiao^19^ and Wang,^24^ and Satman^20^ sampled people ≥20 years.

Sparso^21^ used the WHO thresholds for IFG and so was only synthesised for iIGT, Wang^24^ assessed glycaemia in men only, and Qiao^19^ did not assess combined IGT/IFG in either sex. Only one study, Tutuncu,^23^ reported data on the prevalence of type 2 diabetes diagnosis with isolated glucose states and is reported narratively. The characteristics of the included studies are described in Table 2.

The quality of the meta‐analysed studies was high, except for one which was moderate^22^ due to representativeness of the target population, unclear random selection, high risk of non‐response bias and unclear reporting of numerators/denominators of interest.

### 3.2 Prevalence of iIGT

**FIGURE 2:**
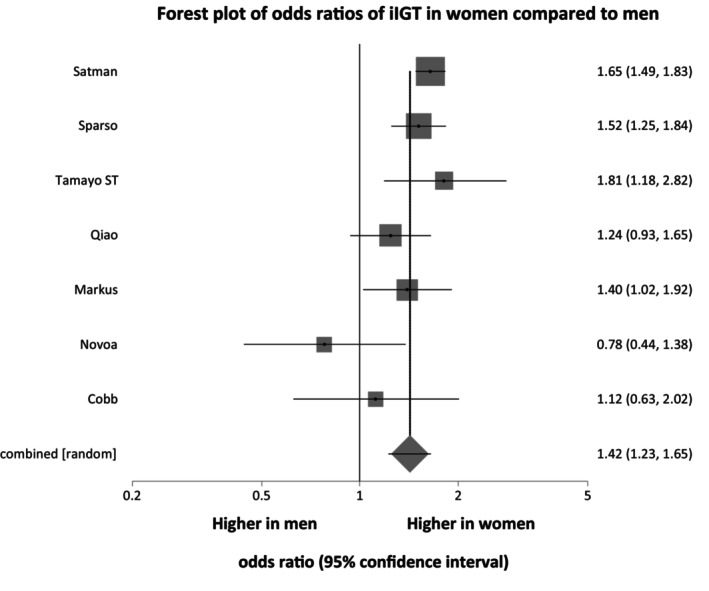
Odds ratio of iIGT in women compared with men.

The pooled prevalence of iIGT in women was 8% [95% CI: 7%, 9%] with considerable heterogeneity, *I*_2_ = 85% [95% CI = 68.6%, 91%] (Figure 1, ESM 2). The pooled prevalence of iIGT in men was 5% [95% CI: 4%, 7%] with considerable heterogeneity, *I* ^2^ = 93.4% [95% CI = 89.8%, 95.3%] (Figure 2, ESM 2).

#### 3.2.1 Subgroup analyses

Removing Satman^20^ (conducted in Turkey) did not have a substantial effect on the size of the pooled prevalences but did result in lower heterogeneity for the prevalence of iIGT in women. Prevalence of iIGT in women 7% [95% CI: 6%, 8%] *I*_2_ = 60.8% [95% CI = 0%, 81.9%] (Table  4, ESM 2).

Sensitivity analyses did not have a substantial effect on the size of the pooled prevalences. Removing Sparso,^21^ and analysing only studies using the ADA threshold (<5.6 mmol/L) to define normal FPG resulted in a pooled prevalence of iIGT in men of 6% [95% CI: 4%, 7%] *I*_2_ = 94.3% [95% CI = 91.3%, 95.9%] (Table  4, ESM 2).

#### 3.2.2 Meta‐analysis of odds ratios comparing the prevalence of iIGT in women and men

The pooled odds of iIGT was 42% [95% CI: 23%, 65%] higher in women than in men, Chi^2^ = 21.53 (df = 1) *p* < 0.0001, *I*_2_ = 52.8% [95% CI = 0%, 78%], Figure 2.

#### 3.2.3 Subgroup analyses

Removing Satman^20^ (conducted in Turkey) did not have a substantial effect on the pooled odds ratio but resulted in lower heterogeneity, with an odds ratio of 1.35 (1.13, 1.60), and an *I* ^2^ of 39.2% [95% CI: 0%, 74.7%]. (Table 4, ESM 2).

**TABLE 4:** Hoy risk of bias assessment.

Criteria	Cobb	Markus	Novoa	Qiao	Satman	Sparso	Tamayo	Tutuncu	Wang
Was the study's target population a close representation of the national population?									
Was the sampling frame a true or close representation of the target population?									
Was some form of random selection used to select the sample or was a census undertaken?	Unclear					Unclear			
Was the likelihood of the non‐response bias minimal?									
Were data collected directly from the subjects?									
Was an acceptable case definition used in the study?								Unclear	
Was the study instrument that measured the parameter of interest shown to have validity and reliability?									
Was the same mode of data collection used for all subjects?									
Was the length of the shortest prevalence period for the parameter of interest appropriate?									
Were the numerators and the denominators for the parameter of interest appropriate?					Unclear		Unclear		
Summary of the overall risk of study bias	8	9	9	10	9	8	7	9	10
	High risk of bias			Moderate risk of bias			Low risk of bias		

Removing Sparso,^21^ and analysing only studies using the ADA threshold (<5.6 mmol/L) to define normal FPG, resulted in a pooled odds ratio of 1.37 (1.12, 1.68), *I*_2_ = 60.6% [95% CI: 0%, 81.9%]. (Table 4, ESM 2).

Restricting the analysis to recent data collection (2005–2012) resulted in a pooled odds ratio of 1.63 [95% CI: 1.48, 1.79] for iIGT in women compared with men with no heterogeneity *I* ^2^ = 0% [95% CI: 0%, 72.9%]. Other analyses did not have a substantial effect on the size and direction of the pooled odds ratios. (Table 4, ESM 2).

### 3.3 Prevalence of iIFG

**FIGURE 3:**
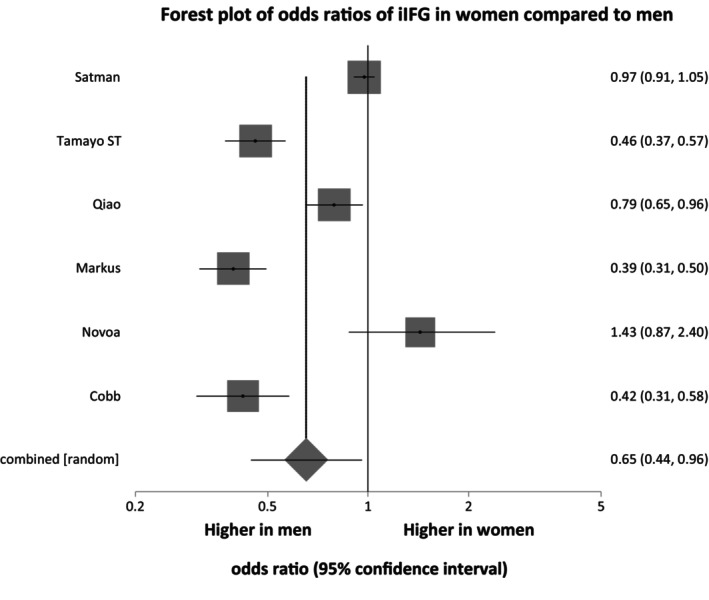
Odds ratio of iIFG in women compared with men.

The pooled prevalence of iIFG in women was 15% [95% CI: 12%, 18%] with considerable heterogeneity, *I* ^2^ = 93.6% [95% CI = 89.4%, 95.7%] (Figure 3, ESM 2). The pooled prevalence of iIFG in men was 24% [95% CI: 13%, 38%] with considerable heterogeneity, *I* ^2^ = 99.8% [95% CI: 99.7%, 99.8%] (Figure 4, ESM 2).

#### 3.3.1 Subgroup analyses

Sensitivity analyses had no substantial effect on the size of the pooled prevalences or heterogeneity (Table 4, ESM 2).

#### 3.3.2 Meta‐analysis of odds ratios comparing the prevalence of iIFG in women and men

The pooled odds of iIFG was 35% [95% CI: 44%, 96%] lower in women than in men, Chi^2^ = 4.78 (df = 1), *p* = 0.0287, *I*_2_ = 95.7% [95% CI = 93.6%, 96.9%], Figure 3.

#### 3.3.3 Subgroup analyses

Removing Satman^20^ (conducted in Turkey) did not have a substantial effect on the pooled odds ratio, 0.59 (0.41, 0.86), *I*_2_ = 90.8% [95% CI: 81.1%, 94.4%]. (Table 4, ESM 2).

Restricting analysis to recent data collection (2005–2012) resulted in a pooled odds ratio of 0.56 [95% CI: 0.30, 1.07] for iIFG in women compared with men with considerable heterogeneity 97.9% [95% CI: 96.6%, 98.5%]. Other analyses did not have a substantial effect on the size and direction of the pooled odds ratios (Table 4, ESM 2).

### 3.4 Prevalence of IGT/IFG

The pooled prevalence of IGT/IFG in women was 7% [5%, 9%], *I*_2_ = 94.1% [95% CI = 89.7%, 96.1%] (Figure 5, ESM 2). The pooled prevalence of IGT/IFG in men was 8% [95% CI = 6%, 12%], *I*_2_ = 97.8% [95% CI = 97%, 98.2%] (Figure 6, ESM 2).

#### 3.4.1 Subgroup analyses

Removing Satman^20^ (conducted in Turkey) did not have a substantial effect on the size of the pooled prevalence, 6% (4%, 8%), *I*_2_ = 83.8% [95% CI: 44.2%, 92%] (Table 4, ESM 2).

Sensitivity analyses did not have a substantial effect on the size of the pooled prevalences. (Table 4, ESM 2).

#### 3.4.2 Odds ratio meta‐analysis for IGT/IFG

There was no difference in the prevalence of combined IGT/IFG between women and men, albeit with considerable uncertainty (pooled odds ratio: 0.85 [95% CI: 0.46, 1.57]) and heterogeneity (*I*_2_ = 95.5% [92.8%, 96.9%]) (Figure 7, ESM 2).

#### 3.4.3 Subgroup analyses

Removing Satman from the analysis resulted in a pooled odds ratio of 0.67 [95% CI: 0.55, 0.81] for IGT/IFG in women compared with men.

Sensitivity analyses did not have a substantial effect on the size and direction of the pooled odds ratios. (Table 4, ESM 2).

### 3.5 Diagnosis of type 2 diabetes with iIGT

Only Tutuncu^23^ reported on the diagnosis of type 2 diabetes by different states (isolated elevated fasting or post‐prandial glucose values or both). Prevalence of type 2 diabetes characterised by isolated elevated post‐prandial glucose was 3.5% [95% CI: 3.2, 3.8%] in women and 2.1% [95% CI: 1.9%, 2.5%]. Prevalence of type 2 diabetes characterised by isolated elevated fasting glucose was 2.2% in both women and men (95% CI in women: [2%, 2.5%] and in men [1.8%, 2.5%]).

Compared with men, women had higher odds of having type 2 diabetes characterized by isolated raised post‐prandial glucose values, 1.6 [95% CI: 1.4, 2.0] The odds ratio of type 2 diabetes diagnosed by isolated raised fasting glucose value: 1.05 [95% CI: 0.87, 1.28] or by HbA_1c_ 0.8 (95% CI: 0.7, 1.0) in women compared with men was not significant.

### 3.6 Certainty of evidence

The certainty of evidence for prevalence was moderate, and for odds ratios was moderate or high, Table 3.

## 4 DISCUSSION

We found that iIGT is more common in women and iIFG is more common in men, whereas the prevalence of combined IGT/IFG is similar in both sexes. Translating the pooled prevalences to the European population would equate to approximately 28 million women and 16 million men with iIGT and 52 million women and 78 million men with iIFG. The pooled odds of iIGT were 42% higher in women and that of iIFG was 35% lower than in men. There was moderate certainty evidence for pooled prevalence estimates and high certainty evidence for comparisons of iIGT and iIFG between sexes. These results confirm a sex difference in isolated IH states and raise concerns about the role of sex in the underdiagnosis and undertreatment of IH and type 2 diabetes.

### 4.1 Strengths and limitations

Meta‐analysis produced a robust estimate of the sex differences in prevalence of isolated IH states using the highly sensitive ADA threshold for fasting glucose. The results have important implications for clinical policy and practice, the interpretation of previous research regarding IH ascertainment and the planning of future research.

However, there are three key limitations to this study. First, the inclusion of studies in the meta‐analysis was impacted by the paucity of sex‐stratified studies assessing isolated IH states. During full‐text screening, 15 studies were excluded due to a lack of sex‐stratified data. This structural limitation of the evidence base is gradually being addressed by initiatives in the UK,^32^ EU,^33^ US^34^ and Canada^35^ that require researchers to account for sex in study proposals and reporting. In the meantime, the development of guidelines for identifying IH differentially among men and women will be limited by the lack of appropriate evidence.

Second, we found that even studies completed within the last 10 years included cohort data from at least 15 years ago and that the oldest data introduced heterogeneity into the results. Sensitivity analysis restricted to the most recent data collection (2005–2012) resulted in increased odds of iIGT in women with 0% heterogeneity. There have been substantial secular changes in obesity and lifestyle that are likely to influence the clinical heterogeneity of populations over time.^36^ Therefore, the pooled prevalences may not be representative of the current population.

Finally, only one of the included studies used the WHO thresholds for IFG; the remaining studies used the ADA thresholds; therefore, it was not possible to produce estimates stratified by different thresholds. This limits the external validity of this review in settings that use the higher WHO threshold.

### 4.2 Results in context

This is the first systematic review and meta‐analysis to estimate the sex‐stratified prevalence of isolated IH states in European populations. The DECODE group conducted an age and sex specific analysis in 2003^12^ and found a similar pattern of results using the WHO 1999 fasting plasma glucose thresholds (≥6.10 mmol/L).

The studies synthesised in the meta‐analyses were of low risk of bias with no evidence of publication bias. Two studies undertook cohort analysis of populations that were not representative of the national population. Of these, Tamayo^22^ was conducted in a region with a high prevalence of type 2 diabetes; this may explain why this study reported the highest estimate of IGT/IFG prevalence in men and the second‐highest in women (Figures 5 and 6, ESM 2). Four studies^16^, ^17^, ^21^, ^22^ were at risk of non‐response bias. Notably, Sparso^21^ found a sex difference in the participation rate, whereby younger women were more likely to participate than younger men; therefore, iIGT prevalence in men may be underestimated in this study. None of the other three studies assessed sex differences in participation rate, and so we were unable to assess how differences in non‐response bias in women compared with men have affected the accuracy of the pooled estimates.

Heterogeneity in the odds ratio of iIGT for women compared with men was reduced by restricting the analysis to more recent data collection (2005–2012). Whilst included studies consistently used the same glycaemic thresholds and standardised OGTT (Table 1), assay method was not reported in four studies, and blood sampling was reported as plasma, capillary and serum in each of three studies, and not reported in five. Consequently, there was insufficient data to assess the clinical heterogeneity associated with these two factors.

Additionally, a cohort study^36^ of this period shows that secular increases in mid‐adulthood waist circumference were greater between 1990–2003 and 2003–2018, particularly in women. Subgroup analyses of this latter period of data collection in our review found that the odds of iIGT in women were even greater, and this reduced heterogeneity to zero. In contrast, the odds of iIFG in women were not significantly different from those in men. Thus, sex‐specific patterns of adiposity and associated secular changes over time may be important considerations for future studies evaluating IH phenotypes and risk.

The risk of type 2 diabetes in women varies over the life course, and this dynamic may be relevant for risk identification. However, only two studies eligible for the meta‐analysis stratified their results by age, Tamayo stratified by decade and Satman by quintennium. In the absence of strata denominators or estimate confidence intervals for Satman, it was not possible to meta‐analyse these data. Consequently, we cannot assess sex‐specific variation in prevalence across the life course. In DECODE,^12^ the prevalence of iIGT was higher in women than in men in each decade from 30 to 89 years, and the difference increased with age. This was broadly echoed by Tamayo's data,^22^ though they assessed people >40 years. In contrast, Satman found that the difference in iIGT prevalence between women and men was wider between 20 and 50 years, then reduced. In DECODE,^37^ iIFG was more common in men until 70 to 79 years, when the prevalence in women overtook it. Tamayo^22^ found that the difference in iIFG prevalence between men and women was substantial up to 60 years, after which it narrowed, though iIFG remained more common in men. The prevalence of iIFG in Satman^20^ was similar in women and men across the life course. Thus, there is a demonstrable intersection between sex and age when estimating the prevalence of IH states that are likely to be associated with post‐menopausal metabolic alterations and may be exacerbated over time by the sex‐specific secular increases in adiposity described above.

Another important consideration is that sexual dimorphism in gut absorption rates, height and body surface area may influence OGTT results. Height is inversely associated with 2‐h post‐75 g load OGTT plasma glucose levels in individuals with isolated IGT.^38^, ^39^ Yet, normoglycaemic people can dispose of varying glucose loads in a very similar manner.^40^ Comparing 50 g and 100 g loads, the mean difference in glucose is 54 mg/dL in people with IGT and only 6 mg/dL in normoglycaemic people.^40^, ^41^ In individuals with IGT, shorter stature is therefore associated with a greater glucose excursion and slower absorption, and this is likely to affect women more than men.^39^, ^42^, ^43^ The sex difference in iIGT prevalence may therefore be acting as a proxy for sexual dimorphism in height. However, the diagnostic threshold for type 2 diabetes was originally defined in sex‐stratified distributions by identifying the cut point between normal and impaired 2‐h post‐load glucose distributions.^44^ Cut points were lower for women than for men in each decile; however, the chosen threshold was the lowest cut point for both men (20–29 and 50+ years) and women (20–29 years).^44^ Thus, concerns about the confounding role of height on diabetes diagnosis and prevalence may be unwarranted.

### 4.3 Clinical relevance

Sex differences in the prevalence of isolated IH states have implications for risk identification in primary care. Clinicians need to be aware that the tests commonly used in primary care are not clinically interchangeable and identify particular pathophysiologically different groups. HbA_1c_ and fasting glucose are likely to miss people with iIGT, and women may be particularly disadvantaged by this process and remain undiagnosed. A 3% difference in the iIGT prevalence between European women and men equates to 11.5 million more women than men with iIGT who are likely to remain undiagnosed and untreated in settings where HbA_1c_ and FPG are utilised for IH assessment.

In many countries, clinicians are advised to use HbA_1c_ to identify IH and type 2 diabetes in primary care, and it is perhaps preferred over FPG, as it can be measured without the patient fasting and at any time.^45^, ^46^, ^47^ However, HbA_1c_ is neither sensitive nor specific for identifying IFG and IGT.^9^ It has been estimated that HbA_1c_ will miss 50% of people with type 2 diabetes (based on OGTT) and will identify a different subgroup of patients than those identified via FPG or OGTT.^45^ This may be explained by variation in the relative contributions of post‐prandial and fasting glucose across the HbA_1c_ range.^48^ Post‐prandial glucose contributes the highest amount in the lowest quintile of HbA_1c_ and progressively decreases, whereas the converse is true for fasting glucose.^49^ Thus, it potentially underdiagnoses iIFG in the lowest quintile and iIGT at the highest.

HbA_1c_ particularly underestimates FPG in men.^50^ In our meta‐analysis, the pooled prevalence of iIFG is substantially higher in men. Therefore, if clinicians preferentially use HbA_1c_ rather than FPG to identify high‐risk individuals, this may result in a higher rate of underdiagnosis in men and undertreatment in people with iIFG overall.

With respect to the accuracy of HbA_1c_ in identifying hyperglycaemia in women, there is evidence that HbA_1c_ is, on average, 1.6 mmol/mol lower in healthy premenopausal women than in men, perhaps due to a shorter erythrocyte survival rate associated with menstruation.^51^ This may affect the sensitivity of HbA_1c_ in identifying IH in women, resulting in underdiagnosis and undertreatment. Conversely, women have higher rates of iron deficiency and anaemia that are, in turn, associated with higher odds of elevated HbA_1c_.^52^ Thus, its specificity in identifying normoglycaemic women may also be lower. The prevalence of IH states is likely to differ significantly across settings that use the WHO thresholds. Barry et al.^9^ assessed the prevalence of isolated IH states and found that the overall prevalences of iIFG and iIGT (ADA threshold) were 25% and 6%, respectively. These rates are very similar to the prevalences we found in men. However, when using the WHO thresholds, they estimated a prevalence of 4.7% for iIFG and 24% for iIGT. Settings using WHO thresholds are therefore likely to classify a much lower proportion of men with iIFG and a higher proportion of women with iIGT. The impact of these differences in thresholds will also vary depending on local testing policies. Where local policies permit, a combination of OGTT yielding fasting and 2 h post‐load glucose with HbA1c would support equitable sex‐specific risk assessment.

These issues with the identification of different IH phenotypes in primary care are likely to result in inequitable rates of underdiagnosis and undertreatment with consequences for the risk of diabetes and its complications. The relative risk of T2D with iIGT is estimated at 5.52, and that of iIFG is 7.53. Furthermore, patients with IGT have elevated all‐cause mortality, coronary heart disease and stroke^53^ than those with IFG. Finally, women, but not men with iIGT, have a significantly raised risk of cardiovascular events and fatal CVD.^54^ A diagnostic pathway that is likely to provide a later diagnosis for people with iIGT and women in particular will therefore leave them at greater risk of macrovascular complications.

### 4.4 Future research

Future research should avoid treating different methods of IH ascertainment as interchangeable. An OGTT and HbA_1c_ should be conducted to identify IH subgroups that can be assessed as predictor variables for diabetes and its complications. If only one means of ascertainment is possible, the results should be interpreted with care and limits to the external validity of the results in considering other IH subgroups should be acknowledged. Researchers should move away from controlling for sex as a confounding variable in statistical models and instead treat it as an effect modifier. At a minimum, all data and analyses should be reported in a sex‐stratified manner, in addition to the main analyses, as this can contribute to future meta‐analyses. Arguably, re‐analysis of existing datasets could provide rapid insight into the intersecting role of sex and IH state in the aetiology of diabetes. Addressing the lag in access to data for individual participant data for meta‐analysis is therefore a priority for evaluating sex differences.

Future studies should revisit the validation of risk tools and the accuracy of diagnostic testing using data and analyses stratified by both sex and IH state. We found only one study evaluating subgroups of type 2 diabetes diagnosed by different states (isolated elevated fasting or postprandial glucose values, or both). Therefore, further analysis evaluating the prevalence of subgroups by sex in European populations is warranted.

Research evaluating the intersection between IH states and sex may provide important information for investigating sex‐specific disparities in the risk of diabetic complications and comorbidities. For example, women transition from IH to type 2 diabetes with a poorer cardiovascular risk profile than men^55^ and subsequently have a 58% and 30% greater risk of CHD and CVD mortality, respectively.^56^ Furthermore, the relative risk of stroke is higher in women, independent of sex differences in other cardiovascular risk factors, including BMI, cholesterol and systolic blood pressure.^57^ Clarity on the sex‐specific incidence of macrovascular and microvascular complications associated with different hyperglycaemic phenotypes is therefore of key importance. The risk of macrovascular disease associated with IGT is dose‐dependent^58^, ^59^, ^60^ and greater than that of IFG.^53^, ^61^ Compared with men with iIGT, women with iIGT are on average shorter, have poorer gut absorption of glucose^42^, ^62^ and higher levels of postprandial glucose. A sex‐stratified analysis of the association between iIGT and the risk of macrovascular disease is clearly warranted.

## 5 CONCLUSIONS

There are sex‐specific disparities in the prevalence of different isolated IH states in European populations, which may contribute to underdiagnosis and undertreatment in primary care. These disparities may introduce ascertainment bias into IH and type 2 diabetes research and policy. Strategies to identify those at high risk of type 2 diabetes may be more effective and equitable if testing procedures are sex‐stratified.

## AUTHOR CONTRIBUTIONS

LC made substantial contributions to the conception and design of the study, the acquisition of data and the analysis and interpretation of the data. She drafted the article and gave final approval for this version to be published. She agreed to be accountable for all aspects of the work in ensuring that questions related to the accuracy or integrity of any part of the work are appropriately investigated and resolved. IP made substantial contributions to the acquisition of data, and the analysis and interpretation of the data. He reviewed the article critically for important intellectual content and approved this version to be published. He agreed to be accountable for all aspects of the work in ensuring that questions related to the accuracy or integrity of any part of the work are appropriately investigated and resolved. CM made substantial contributions to the conception and design of the study, and the analysis and interpretation of the data. She reviewed the article critically for important intellectual content and approved this version to be published. She agreed to be accountable for all aspects of the work in ensuring that questions related to the accuracy or integrity of any part of the work are appropriately investigated and resolved. AA made substantial contributions to the analysis and interpretation of the data. She reviewed the article critically for important intellectual content and approved this version to be published. She agreed to be accountable for all aspects of the work in ensuring that questions related to the accuracy or integrity of any part of the work are appropriately investigated and resolved. SG made substantial contributions to the analysis and interpretation of the data. He reviewed the article critically for important intellectual content and approved this version to be published. He agreed to be accountable for all aspects of the work in ensuring that questions related to the accuracy or integrity of any part of the work are appropriately investigated and resolved.

## FUNDING INFORMATION

This research is supported by the Medical Research Council (MRC) (MC_UU_00006/6) and the NIHR Cambridge Biomedical Research Centre (NIHR203312). The views expressed are those of the authors and not necessarily those of the MRC, NIHR or the Department of Health and Social Care. The University of Cambridge has received salary support in respect of SJG from the NHS in the East of England through the Clinical Academic Reserve. The funders of the study had no role in the design, data collection, data analysis, data interpretation, or writing of the report. For the purpose of open access, the author has applied a Creative Commons Attribution (CC BY) license to any Author Accepted Manuscript version arising from this submission.

## CONFLICT OF INTEREST STATEMENT

None.

## Supporting information


Data S1:

Data S2:

## Data Availability

This study was a re‐analysis of existing data, which is openly available at locations cited in the reference section.
