# Supplementary material for: Sex‐specific differences in the prevalence of intermediate hyperglycaemia states: A systematic review and meta‐analysis
Source: Diabet Med. 2026 Mar 10;43(8):e70293. doi: 10.1111/dme.70293 (PMC13380399; doi:10.1111/dme.70293)
Supplement: Supplementary file 1 — Data S1: [file DME-43-e70293-s002.docx]

# ESM 1

## Protocol

**DESCRIPTION OF THE ISSUE**

Intermediate hyperglycaemia (IH) is a key risk factor for type 2 diabetes that presents in different states: impaired glucose tolerance (IGT), impaired fasting glucose (IFG) and combined IGT/IFG. IGT is more common than IFG, and affects more than 623 million adults globally, with 370 million adults expected to have IFG ^1^. Identification of people with IH is a key component of diabetes prevention programmes.

Tests for glycaemia identify different IH subgroups and, therefore, different people. Though more costly and onerous for patients and practitioners, the oral glucose tolerance test (OGTT) will identify people with either iIFG or iIGT or combined IGT/IFG. The World Health Organisation (WHO) estimates that using fasting plasma glucose alone will miss 30% of cases ^2^. HbA_1c_ is easy to administer in primary care; however, in a meta-analysis, Barry et al. (2017) ^3^ demonstrated that HbA_1c_ is neither sensitive nor specific for identifying IH states.

Sex differences in the prevalence of IGT and IFG have long been recognised ^4,5^ and were described in European populations by the Diabetes Epidemiology: Collaborative Analysis of Diagnostic Criteria in Europe (DECODE) study in 2003 ^6^.

**Why it is important to do this review**

Disparities in the prevalence of glucose states between the sexes may intersect with the variable sensitivity and specificity of tests and result in unequal access to diagnosis and treatment.

**Objective:**

To assess sex-specific prevalences for isolated IGT, isolated IFG, and combined IGT/IFG in cross-sectional and cohort studies of European people.

**Review questions**

1. What are the sex specific prevalences of iIGT, iIFG, combined IFG/IGT and undiagnosed type 2 diabetes (T2DM) when the oral glucose tolerance test (OGTT) is used to diagnose intermediate hyperglycaemia (IH) or T2DM characterised by isolated hyperglycaemic states, in European cohort and cross-sectional studies?
2. Is there a significant sex difference in the odds of iIGT, iIFG, combined IFG/IGT or T2DM (characterised by isolated hyperglycaemic states)?
3. If there is a sex difference, what are the implications for the health equity of diabetes screening and diagnosis protocols?

**METHODS**

**SEARCH STRATEGY**
PubMed, Cochrane, Embase and CINAHL will be searched from inception to 30th October 2023. Authors of included studies will be contacted to identify additional unpublished studies. Forward and backward citation searches of included studies will also be conducted. The search will not be restricted by publication status, language or date. Searches will be conducted by LC.
Search terms
A PICOS concept map identified that MeSH terms were not specific enough to identify studies for inclusion. Keywords will be used to ensure that the outcome measures needed to identify the prevalence of the different phenotypes are included in the study.
The search terms are iIGT, iIFG, and iHbA_1c_ plus all possible versions of these terms.

**Study design**

**Inclusion**
• Cohort studies
• Cross-sectional studies
**Exclusion**
• Intervention studies
• Case studies/series

**ELIGIBILITY CRITERIA**

**Population**

**Inclusion criteria**
• European population
• Adult population 18-75
**Exclusion criteria**
• Children
• Non-European population
• Elderly population
• Pregnant women or women who have experienced gestational diabetes
• Populations with existing disease
• Populations who have been identified as relatives of diabetic patients
• Populations identified as being at high risk of T2DM
• Populations with type 1 diabetes

**Exposure(s)**

All studies must have measured 2-hour post-load (75g) glucose tolerance and fasting glucose levels to accurately classify participants as having normal glucose tolerance, iIGT, iIFG, IGT/IFG or T2DM (with iIGT or iIFG).
The OGTT has several different permutations; only studies which have a 2-hour value will be included in the review and meta-analysis to ensure consistency with the U.K. guidelines.
The studies must have used the WHO or ADA thresholds for classifying iIGT and iIFG.
Where available, collating HbA1c or iHbA1c data will give further opportunity for evaluating the complete NICE protocol for prediabetes and diabetes diagnosis and consideration of sex specific patterns in the relationship between average glucose and iIFG and iIGT.
**Inclusion criteria**
• Studies with iIGT, iIFG, IFG/IGT, T2Dm prevalence data
• Studies with raw participant data from which prevalence and odds ratios can be calculated.
**Exclusion**
• Studies with no iIGT, iIFG, IFG/IGT, T2Dm prevalence data
• Studies with no raw participant data from which prevalence and odds ratios can be calculated

**Comparator(s) or control(s)**

Not applicable

**Context**

**Inclusion**
Studies conducted in European populations, which will therefore have similar ancestry to the UK, and are likely to have similar prevalence of IH and diabetes.
**Exclusion**
Non-European populations, for example, populations of Asian ancestry, are likely to have different IH or diabetes prevalence and would therefore weaken the external validity of the meta-analysis in considering the health equity of UK populations.

**OUTCOMES TO BE ANALYSED**

**Main outcomes**

Primary outcome measures:
Pooled crude Prevalence of iIGT, iIFG and IGT/IFG
Pooled crude Prevalence of iIGT, iIFG and IGT/IFG in women
Pooled crude Prevalence of iIGT, iIFG and IGT/IFG in men
Pooled prevalence of T2DM (with isolated increased 2-hour glucose [OGTT]) in women compared to men
Pooled prevalence of T2DM (with isolated increased fasting glucose) in women compared to men
Pooled prevalence of T2DM (combined increased 2-hour and fasting glucose) in women compared to men

**Measures of effect**

Odds ratio of iIGT in women compared to men
Odds ratio of iIFG in women compared to men
Odds ratio of IGT/IFG in women compared to men
Odds ratio of T2DM (with isolated increased 2-hour glucose [OGTT]) in women compared to men
Odds ratio of T2DM (with isolated increased fasting glucose) in women compared to men
Odds ratio of T2DM (combined increased 2-hour and fasting glucose) in women compared to men

**DATA COLLECTION PROCESS**

**Selection of studies**
The complete search will be uploaded to Mendeley reference manager and then to Covidence online systematic review management software. Initial screening of titles and abstracts will be conducted by LC and IP, with disagreements resolved through discussion. Studies which clearly do not meet the inclusion criteria will be removed.

Full text versions of the studies which pass this initial screening will then be screened using a predetermined screening tool based on the inclusion/exclusion criteria. Full text screening will be conducted by LC and IP, with disagreements resolved through discussion. A record of reasons for exclusion for each study will be kept and a PRISMA flow chart will be completed. Articles in a language other than English will be translated.

Multiple reports of the same study will be collated in preparation for data extraction.
Data extraction and management

A data extraction tool (created in Excel) will be piloted and then used by LC to extract outcome data and checked by IP. Disagreements will be resolved by discussion.

The data extraction tool includes:
Publication type, country
Type of study
Participants age, social or cultural characteristics, definition of geographic boundary
Type of screening test and the protocol followed
Outcome measures and the diagnostic thresholds used
Study aims, social context, date of cross-sectional study or cohort baseline assessment date
Recruitment methods
Inclusion/exclusion criteria for participation in study
Representativeness of the sample for our target population
Statistical methods used and appropriateness of these methods
Participation rate/refusal to participate rate/enrolment outcomes
Missing data
Sex, Age, ethnicity sub-groups, weight/BMI, waist circumference, other sociodemographic PROGRESS categories included
Results: no. of men/women in each ethnic group for each outcome, crude and adjusted prevalence data for each subset
All potential moderators/confounders of the study outcomes are included in the extraction form.

**RISK OF BIAS (QUALITY) ASSESSMENT**

The risk of bias of each study design will be assessed using the JBI critical appraisal checklist, as this is the most relevant for studies which yield prevalence rates. Critical appraisal will be conducted independently by LC and checked by IP, with disagreement resolved by discussion. Justification of risk of bias judgements will be included in the risk of bias tables using direct quotes from the study paper. The descriptors for each item on the risk of bias form will be used by each reviewer.
Appropriateness of sample frame
Appropriateness of sampling of participants
Adequacy of sample size
Sufficiently detailed description of subjects and setting
Sufficient coverage of the identified sample in the data analysis
Standardisation of criteria and methodology for measuring iIGT/iIFG
Appropriateness of statistical analysis
Adequacy of response rate
Assessment of reporting biases and small study effects:
Publication bias is possible in cohort studies and will be assessed with a contour-enhanced funnel plot if there are more than ten studies. If there are fewer than ten, the guidance in the Cochrane Handbook will be followed.
The funnel plot may also identify small study effects, which will then be followed up with a sensitivity analysis.

**PLANNED DATA SYNTHESIS**

**Strategy for data synthesis**

Meta-analysis will be carried out using a random effects model. To pool proportions of iIGT, iIFG and IFG/IGT, the data will be transformed using the Freeman-Tukey transformation.
Prevalence with 95% confidence intervals will be calculated for overall iIFG, iIGT, combined IFG/IGT, T2DM diagnosed by iIGT, iIFG or HbA1c alone. Sex specific prevalences will be calculated for each of these prediabetes/diabetes phenotypes.
Pooled odds ratio (with 95% confidence intervals) of iIGT, iIFG, IFG/IGT and T2DM in women compared to men will be calculated for a subset of studies reporting on both sexes.
**Assessment of heterogeneity**
Consideration will be given to the heterogeneity of populations to be included in the meta-analysis. Heterogeneity will be evaluated through visual inspection of a forest plot and consideration of the clinical or methodological heterogeneity of the included studies. The I² statistic will also be calculated and evaluated in conjunction with the causes of variation between studies. Subgroup analysis of sex and age group will be conducted to investigate heterogenous results and provide an estimate of the effect of these groups. These analyses will be used to decide whether a meta-analysis is possible.

**Quality of the evidence assessment**
The Grading of Recommendations, Assessment, Development and Evaluation (GRADE) approach will be used to rate the quality of the evidence and assess the level of certainty in prevalence estimates.

**References**

1. IDF Diabetes Atlas 10th Edition. https://diabetesatlas.org/data/en/.

2. *Definition and Diagnosis of Diabetes Mellitus and Intermediate Hyperglycemia Report of a WHO/IDF ConsultatIon*. (2006).

3. Barry, E. *et al.* Efficacy and effectiveness of screen and treat policies in prevention of type 2 diabetes: Systematic review and meta-analysis of screening tests and interventions. *BMJ (Online)* 356, (2017).

4. Kautzky-Willer, A., Leutner, M. & Harreiter, J. Sex differences in type 2 diabetes. *Diabetologia* 66, 986–1002 (2023).

5. Kautzky-Willer, A., Harreiter, J. & Pacini, G. Sex and gender differences in risk, pathophysiology and complications of type 2 diabetes mellitus. *Endocr Rev* 37, 278–316 (2016).

6. THE DECODE STUDY GROUP. Age and Sex specific prevalences of Diabetes and Impaired Glucose Regulation in 13 European Cohorts. *Diabetes Care* 26, 61–69 (2003).
