# Supplementary material for: Sex‐specific differences in the prevalence of intermediate hyperglycaemia states: A systematic review and meta‐analysis
Source: Diabet Med. 2026 Mar 10;43(8):e70293. doi: 10.1111/dme.70293 (PMC13380399; doi:10.1111/dme.70293)
Supplement: Supplementary file 2 — Data S2: [file DME-43-e70293-s001.docx]

## Electronic Supplementary Materials 2

## PICOS concept map

| RQ: What are the sex specific prevalences of isolated impaired glucose tolerance, (iIGT), isolated fasting plasma glucose (iIFG), isolated HbA1c (iHbA1c) and Type 2 Diabetes diagnosis (using OGTT) in European populations. | | | | | | |
| --- | --- | --- | --- | --- | --- | --- |
| P | I | C | Outcome | O | O | S |
| Adults aged 18 to 74 | Glucose tolerance test | Fasting glucose test or HbA1c | i-IGT  iIGT  isolated IGT  isolated impaired glucose tolerance | i-IFPG  iIFG  isolated IFG  isolated impaired fasting  glucose  isolated fasting hyperglycemia | i-HbA1c  iHbA1c  isolated HbA1c  isolated A1c | Prevalence studies, baseline data of cohort and intervention studies. |
| No restriction to maximise sensitivity, European focus if sufficient studies | Too broad for specificity | Too broad for specificity | Keyword searches only as these are not MeSH terms | Keyword searches only as these are not MeSH terms | Keyword searches only as these are not MeSH terms | No restriction to maximise sensitivity |

Table 1 PICOS concept map

## Sample search strategy

| **Embase (OVID)**  1. "i-IGT".ab,kw,ti.  2. "iIGT".ab,kw,ti.  3. "isolated IGT".ab,kw,ti.  4. "isolated impaired glucose tolerance".ab,kw,ti.  5. 1 or 2 or 3 or 4  6. "i-IFG".ab,kw,ti.  7. "iIFG".ab,kw,ti.  8. "isolated IFG".ab,kw,ti.  9. "isolated impaired fasting glucose".ab,kw,ti.  10. "isolated fasting hyperglycemia".ab,kw,ti.  11. 6 or 7 or 8 or 9 or 10  12. "i-HbA1c".ab,kw,ti.  13. "iHbA1c".ab,kw,ti.  14. "isolated A1c".ab,kw,ti.  15. "isolated HbA1c".ab,kw,ti.  16. 12 or 13 or 14 or 15  17. 11 or 16  18. 5 and 17 |
| --- |
| **Medline (OVID)**  1. "i-IGT".ab,kw,ti.  2. "iIGT".ab,kw,ti.  3. "isolated IGT".ab,kw,ti.  4. "isolated impaired glucose tolerance".ab,kw,ti.  5. 1 or 2 or 3 or 4  6. "i-IFG".ab,kw,ti.  7. "iIFG".ab,kw,ti.  8. "isolated IFG".ab,kw,ti.  9. "isolated impaired fasting glucose".ab,kw,ti.  10. "isolated fasting hyperglycemia".ab,kw,ti.  11. 6 or 7 or 8 or 9 or 10  12. "i-HbA1c".ab,kw,ti.  13. "iHbA1c".ab,kw,ti.  14. "isolated A1c".ab,kw,ti.  15. "isolated HbA1c".ab,kw,ti.  16. 12 or 13 or 14 or 15  17. 11 or 16  18. 5 and 17 |
| **Cinahl**  S1 "i-IGT" OR "iIGT" OR "isolated IGT" OR "isolated impaired glucose tolerance"  S2  "i-IFG" OR "iIFG" OR "isolated IFG" OR "isolated impaired fasting glucose" OR "isolated fasting hyperglycemia"  S3 "isolated A1c" OR "isolated HbA1c" OR "i-HbA1c"  S4 S2 OR S3  S5 S1 AND S4 |
| **Cochrane Database of Systematic Reviews**  #1: ("i-IGT" OR "iIGT" OR "isolated IGT" OR "isolated impaired glucose tolerance")  #2: ("i-IFG" OR "iIFG" OR "isolated IFG" OR "isolated impaired fasting glucose" OR "isolated fasting hyperglycemia")  #3: ("i-HbA1c" OR "iHbA1c" OR "isolated HbA1c" OR "isolated HbA1c" OR "isolated A1c")  #4: #1 OR #2 OR #3 |

Table 2 Searches

## **Excluded studies**

| **Unable to access full text from authors**^1–4^ |
| --- |
| Chen, G. *et al.* Comparison of Insulin Resistance and beta-Cell Dysfunction Between the Young and the Elderly in Normal Glucose Tolerance and Prediabetes Population: A Prospective Study. *Hormone and Metabolic Research* **49**, 135–141 (2017).   1. Kato, K. *et al.* Elevated C-reactive Protein Levels Independently Predict the Development of Prediabetes Markers in Subjects with Normal Glucose Regulation. *Exp Clin Endocrinol Diabetes* **129**, 289–295 (2021).   7. Hutchinson, M. S. *et al.* Serum 25-Hydroxyvitamin D levels in subjects with reduced glucose tolerance and type 2 diabetes-The Tromso OGTT-Study. *International Journal for Vitamin and Nutrition Research* **81**, 317–327 (2011).  8. Hung, H. C. *et al.* Associations between GDF15 Levels and Pre-Diabetes in Non-Obese Subjects. *Journal of Investigative Medicine* **70**, 79–84 (2022). |
| **Sex stratified analysis not possible**^5–19^ |
| . Gylling, H. *et al.* Cholesterol metabolism in relation to glucose tolerance in a large Finnish male population. *Atheroscler Suppl* **10**, (2009).   1. De Vegt F. *et al.* Elevated fasting and postload glucose concentrations: Risk factors for developing type-2 diabetes mellitus; results of the Hoorn study. *Ned Tijdschr Geneeskd* **147**, 1414–1418 (2003).   11. Kohlenberg, J. D. *et al.* Differential contribution of alpha and beta cell dysfunction to impaired fasting glucose and impaired glucose tolerance. *Diabetologia* **66**, 201–212 (2023).  12. Valdes S. *et al.* Prevalence of diabetes and impaired glucose regulation in Spain: Di@bet.es study. *Diabetologia* **54**, S140 (2011).  13. Valdés, S. *et al.* Population-based incidence of type 2 diabetes in northern Spain: The Asturias study. *Diabetes Care* **30**, 2258–2263 (2007).  14. Rathmann W. *et al.* Incidence of Type 2 diabetes in the elderly German population and the effect of clinical and lifestyle risk factors: KORA S4/F4 cohort study. *Diabetic Medicine* **26**, 1212–1219 (2009).  15. Johansen, N. B. *et al.* Effect of regression or progression in glucose tolerance status on aortic stiffness: The ADDITION-PRO study. *Diabetes* **62**, A369–A370 (2013).  16. Linder, K. *et al.* Allele summation of diabetes risk genes predicts impaired glucose tolerance in female and obese individuals. *PLoS One* **7**, e38224 (2012).  17. Naeem, M. *et al.* Association of hepatic steatosis derived from ultrasound and quantitative MRI with prediabetes in the general population. *Sci Rep* **11**, 13276 (2021).  18. Naeem, M. *et al.* Association between hepatic iron overload assessed by magnetic resonance imaging and glucose intolerance states in the general population. *Nutrition, Metabolism and Cardiovascular Diseases* **32**, 1470–1476 (2022).  19. Yip, W. C. Y. Y., Sequeira, I. R. , Plank, L. D. , & Poppitt, S. D., Prevalence of pre-diabetes across ethnicities: A review of impaired fasting glucose (IFG) and impaired glucose tolerance (IGT) for classification of dysglycaemia. *Nutrients* **9**, 1273 (2017).  20. Eleftheriadou, A. *et al.* The prevalence of cardiac autonomic neuropathy in prediabetes: a systematic review. *Diabetologia* **64**, 288–303 (2021).  21. Meisinger, C. *et al.* Retinol-binding protein 4 is associated with prediabetes in adults from the general population: The cooperative health research in the Region of Augsburg (KORA) F4 study. *Diabetes Care* **34**, 1648–1650 (2011).  22. Meisinger C. *et al.* Serum potassium is associated with prediabetes and newly diagnosed diabetes in hypertensive adults from the general population: The KORA F4-Study. *Diabetologia* **56**, 484–491 (2013).  De Simone G. *et al.* Metabolic phenotypes and cardiovascular abnormalities in prediabetes. *J Am Coll Cardiol* **55**, A58 (2010). |
| **Wrong Geographical area**^20–34^ |
| Lorenzo, C. *et al.* Impaired fasting glucose and impaired glucose tolerance have distinct lipoprotein and apolipoprotein changes: The insulin resistance atherosclerosis study. *Journal of Clinical Endocrinology and Metabolism* **98**, 1622–1630 (2013).  25. Nichols, G. A., Arondekar, B. & Herman, W. H. Medical care costs one year after identification of hyperglycemia below the threshold for diabetes. *Med Care* **46**, 287–292 (2008).  26. Ohshita, K. *et al.* Elevated White Blood Cell Count in Subjects with Impaired Glucose Tolerance. *Diabetes Care* **27**, 491–496 (2004).  27. Kim, S. H. & Reaven, G. M. Isolated Impaired Fasting Glucose and Peripheral Insulin SensitivityNot a simple relationship. *Diabetes Care* **31**, 347–352 (2008).  28. Liu, J. *et al.* Reduced Circulating Endothelial Progenitor Cells and Downregulated GTCPH i Pathway Related to Endothelial Dysfunction in Premenopausal Women with Isolated Impaired Glucose Tolerance. *Cardiol Res Pract* **2020**, 1278465 (2020).  29. Festa, A., Lorenzo, C., Wagenknecht, L. E., Hanley, A. J. G. & Haffner, S. M. Lipoproteins measured by conventional methods vs. Nuclear Magnetic Resonance (NMR) spectroscopy and association with glucose tolerance status: The Insulin Resistance Atherosclerosis Study (IRAS). *Diabetes* **60**, A190–A191 (2011).  30. Hong, S. M. *et al.* Glycated albumin is a useful indicator for screening impending diabetes and predicting beta cell dysfunction in the pre-diabetic condition. *Diabetologia* **57**, S123 (2014).  31. Hong, S. M. *et al.* Glycated albumin is a useful indicator for predicting beta cell dysfunction and impending diabetes in prediabetic condition. *Diabetes Res Clin Pract* **106**, S89–S90 (2014).  32. Wang, X. *et al.* A study comparing the prevalence of urinary albumin excretion and microalbuminuria in pre-diabetes subjects. *Zhonghua Nei Ke Za Zhi* **43**, 170–173 (2004).  33. Wang, Z. *et al.* Immunoglobulin e and mast cell proteases are potential risk factors of impaired fasting glucose and impaired glucose tolerance in humans. *Ann Med* **45**, 220–229 (2013).  34. Sasaki, N., Maeda, R., Ozono, R., Nakano, Y. & Higashi, Y. Association of obesity with serum free fatty acid levels in individuals at different stages of prediabetes. *Clin Obes* **12**, e12496 (2022).  35. Marunaka, Y. *et al.* Possibility of Venous Serum Cl- Concentration ([Cl-]s) as a Marker for Human Metabolic Status: Correlation of [Cl-]s to Age, Fasting Blood Sugar (FBS), and Glycated Hemoglobin (HbA1c). *Int J Mol Sci* **22**, (2021).  36. Pankow, J. S. *et al.* Cardiometabolic risk in impaired fasting glucose and impaired glucose tolerance: The atherosclerosis risk in communities study. *Diabetes Care* **30**, 325–331 (2007).  37. Nichols, G. A., Arondekar, B., Herman, W. H., G.A., N. & B., A. Complications of dysglycemia and medical costs associated with nondiabetic hyperglycemia. *Am J Manag Care* **14**, 791–798 (2008).  38. Piche M.E. *et al.* Impaired fasting glucose vs. glucose intolerance in pre-menopausal women: Distinct metabolic entities and cardiovascular risk? *Diabetic Medicine* **21**, 730–737 (2004). |
| **Overlapping studies of the same cohort**^35–40^ |
| Staacakova, A. *et al.* Changes in insulin sensitivity and insulin release in relation to glycemia and glucose tolerance in 6,414 finnish men. *Diabetes* **58**, 1212–1221 (2009).  40. Markus, M. R. P. *et al.* Prediabetes is associated with lower brain gray matter volume in the general population. The Study of Health in Pomerania (SHIP). *Nutrition, Metabolism and Cardiovascular Diseases* **27**, 1114–1122 (2017).  41. Kowall, B. *et al.* Incidence rates of type 2 diabetes in people with impaired fasting glucose (ADA vs. WHO Criteria) and impaired glucose tolerance: Results from an older population (KORA S4/F4/FF4 Study). *Diabetes Care* **42**, E18–E20 (2019).  42. Ziegler, D. *et al.* Increased prevalence of cardiac autonomic dysfunction at different degrees of glucose intolerance in the general population: the KORA S4 survey. *Diabetologia* **58**, 1118–1128 (2015).  43. Meisinger, C. *et al.* Uric acid is more strongly associated with impaired glucose regulation in women than in men from the general population: The KORA F4-study. *PLoS One* **7**, e37180 (2012).  44. Meisinger, C. *et al.* Prevalence of undiagnosed diabetes and impaired glucose regulation in 35-59-year-old individuals in Southern Germany: The KORA F4 study. *Diabetic Medicine* **27**, 360–362 (2010). |
| **Pre-existing hyperglycemia**^41–44^ |
| Aye, M. M. *et al.* Progression of Impaired Glucose Regulation (IGR) to type 2 diabetes. *Diabetic Medicine* **27**, 33 (2010).  2. Giráldez-García, C. *et al.* The heterogeneity of reversion to normoglycemia according to prediabetes type is not explained by lifestyle factors. *Scientific Reports 2021 11:1* **11**, 1–11 (2021).  3. Faerch, K. & Vaag, A. Metabolic inflexibility is a common feature of impaired fasting glycaemia and impaired glucose tolerance. *Acta Diabetol* **48**, 349–353 (2011).  4. Cobb, J. E., Eckhart, A., Adam, K.-P., Pappan, K. & Sinnott, M. Metabolomic characterization of impaired fasting glucose (IFG) and impaired glucose tolerance (IGT). *Diabetes* **63**, A358 (2014). |
| **Pre-existing risk of type 2 diabetes**^45–48^ |
| Engberg, S. *et al.* Differential relationship between physical activity and progression to diabetes by glucose tolerance status: The Inter99 Study. *Diabetologia* **53**, 70–78 (2010).  46. Schmid, V. *et al.* Liver fat content and insulin secretion failure are associated with the long-term success of a lifestyle intervention to prevent type 2 diabetes: Results of the TULIP study. *Diabetologia* **59**, S171 (2016).  47. Tura, A. *et al.* Profiles of glucose metabolism in different prediabetes phenotypes, classified by fasting glycemia, 2-hour OGTT, glycated hemoglobin, and 1-hour OGTT: An IMI DIRECT study. *Diabetes* **70**, 2092–2106 (2021).  48. Lidegaard, L. P. L. P. *et al.* Physical activity energy expenditure vs cardiorespiratory fitness level in impaired glucose metabolism. *Diabetologia* **58**, 2709–2717 (2015). |
| **Systematic reviews**^49–51^ |
| Larsen, M. P. *et al.* Glucagon-Like Peptide 1: A Predictor of Type 2 Diabetes? *J Diabetes Res* **2017**, 7583506 (2017).  50. Liu, Y. *et al.* Evidence From a Systematic Review and Meta-Analysis: Classical Impaired Glucose Tolerance Should Be Divided Into Subgroups of Isolated Impaired Glucose Tolerance and Impaired Glucose Tolerance Combined With Impaired Fasting Glucose, According to the Risk . *Front Endocrinol (Lausanne)* **13**, (2022).  51. Gerstein, H. C. *et al.* Annual incidence and relative risk of diabetes in people with various categories of dysglycemia: A systematic overview and meta-analysis of prospective studies. *Diabetes Research and Clinical Practice* vol. 78 305–312 Preprint at https://doi.org/10.1016/j.diabres.2007.05.004 (2007). |
| **Non-random population**^52,53^ |
| Simonen, P. *et al.* Cholesterol metabolism in relation to glucose tolerance in a Finnish male population. *J Diabetes* **1**, A47 (2009).  53. Faerch, K. *et al.* Impaired fasting glycaemia vs impaired glucose tolerance: Similar impairment of pancreatic alpha and beta cell function but differential roles of incretin hormones and insulin action. *Diabetologia* **51**, 853–861 (2008). |
| **Pre-existing disease**^54,55^ |
| Duran, I. D. *et al.* Irisin levels in the progression of diabetes in sedentary women. *Clin Biochem* **48**, 1268–1272 (2015).  Borel, A. L. *et al.* Visceral, subcutaneous abdominal adiposity and liver fat content distribution in normal glucose tolerance, impaired fasting glucose and/or impaired glucose tolerance. *Int J Obes* **39**, 495–501 (2015). |
| **Gestational diabetes^56^** |
| Sokup, A., Szymanski, M. & Goralczyk, K. Impaired fasting glucose as a marker of heterogeneity of gestational diabetes mellitus. A study of 1025 women living in the region of Kuyavia and Pomerania in Poland. *Endokrynol Pol* **60**, 348–352 (2009). |
| **No baseline data**^57^ |
| Faerch, K. *et al.* Natural history of insulin sensitivity and insulin secretion in the progression from normal glucose tolerance to impaired fasting glycemia and impaired glucose tolerance: The inter99 study. *Diabetes Care* **32**, 439–444 (2009). |
| **Outcome not stratified by iIGT and/or iIFG**^58^ |
| Pitchika, A. *et al.* Hepatic steatosis and hepatic iron overload modify the association of iron markers with glucose metabolism disorders and metabolic syndrome. *Liver International* **41**, 1841–1852 (2021). |
| **Wrong criteria for ascertainment used**^59,60^ |
| 59. de Vegt, F. *et al.* Relation of impaired fasting and postload glucose with incident type 2 diabetes in a Dutch population: The Hoorn study. *J Am Med Assoc* **285**, 2109–2113 (2001).  Iriarte-Campo, V. *et al.* *Incidence of T2DM and the Role of Baseline Glycaemic Status as a Determinant in a Metropolitan Population in Northern Madrid (Spain) - ScienceDirect*. *Diabetes Research and Clinical Practice* vol. 209 111119 (Elsevier, 2024). |

Table 3 Excluded studies

## **Prevalence of iIGT**

## iIGT prevalence in women

Method: Stuart-Ord (inverse double arcsine square root)

Non-combinability of studies

Cochran Q = 40.055672  (df = 6)  P < 0.0001

Moment-based estimate of between studies variance = 0.002487

I_2_ (inconsistency) = 85% (95% CI = 68.6% to 91%)

Random effects (DerSimonian-Laird)

Pooled proportion = 0.07738 (95% CI = 0.066417 to 0.089105)

Bias indicators

Begg-Mazumdar: Kendall's -0.142857  P = 0.5619

Egger: bias = -2.999006 (95% CI = -6.839946 to 0.841933)  P = 0.101

Harbord: bias = -2.886252 (92.5% CI = -5.754049 to -0.018454)  P = 0.0737

0.04

0.05

0.06

0.07

0.08

0.09

0.10

0.11

**Prevalence of iIGT in women**

combined

0.08 (0.07, 0.09)

Cobb

0.06 (0.04, 0.09)

Novoa

0.06 (0.04, 0.09)

Markus

0.08 (0.07, 0.10)

Qiao

0.10 (0.08, 0.11)

Tamayo ST

0.07 (0.05, 0.09)

Satman

0.09 (0.09, 0.10)

Sparso

0.07 (0.06, 0.08)

proportion (95% confidence interval)

Figure 1 Pooled prevalence of iIGT in women

## iIGT prevalence in men

Method: Stuart-Ord (inverse double arcsine square root)

Non-combinability of studies

Cochran Q = 105.626369  (df = 7)  P < 0.0001

Moment-based estimate of between studies variance = 0.005002

I_2_ (inconsistency) = 93.4% (95% CI = 89.8% to 95.3%)

Random effects (DerSimonian-Laird)

Pooled proportion = 0.054377 (95% CI = 0.042929 to 0.067091)

Bias indicators

Begg-Mazumdar: Kendall's 0.214286  P = 0.5484

Egger: bias = 3.174566 (95% CI = -2.108214 to 8.457346)  P = 0.1919

Harbord: bias = 2.705638 (92.5% CI = -3.006316 to 8.417592)  P = 0.3476

0.03

0.04

0.05

0.06

0.07

0.08

0.09

0.10

0.11

**Prevalence of iIGT in men**

combined

0.05 (0.04, 0.07)

Wang

0.03 (0.03, 0.04)

Cobb

0.06 (0.04, 0.08)

Novoa

0.08 (0.05, 0.11)

Markus

0.06 (0.05, 0.07)

Qiao

0.08 (0.06, 0.10)

Tamayo ST

0.04 (0.03, 0.05)

Satman

0.06 (0.05, 0.06)

Sparso

0.05 (0.04, 0.05)

proportion (95% confidence interval)

Figure 2 Pooled prevalence of iIGT in men

## **Prevalence of iIFG**

## iIFG prevalence in women (ADA threshold)

Proportion meta-analysis

 Method: Stuart-Ord (inverse double arcsine square root)

Non-combinability of studies

Cochran Q = 78.22386  (df = 5)  P < 0.0001

Moment-based estimate of between studies variance = 0.008651

I_2_ (inconsistency) = 93.6% (95% CI = 89.4% to 95.7%)

Random effects (DerSimonian-Laird)

Pooled proportion = 0.147303 (95% CI = 0.120574 to 0.176209)

Bias indicators

Begg-Mazumdar: Kendall's 0.066667  P > 0.9999

Egger: bias = 0.054438 (95% CI = -8.298407 to 8.407282)  P = 0.9864

Harbord: bias = 0.095839 (92.5% CI = -6.680385 to 6.872063)  P = 0.9746

0.08

0.10

0.12

0.14

0.16

0.18

0.20

0.22

**Prevalence of iIFG in women (ADA threshold)**

combined

0.15 (0.12, 0.18)

Cobb

0.16 (0.13, 0.20)

Novoa

0.11 (0.09, 0.14)

Markus

0.09 (0.08, 0.11)

Qiao

0.18 (0.16, 0.20)

Tamayo ST

0.20 (0.17, 0.22)

Satman

0.15 (0.14, 0.15)

proportion (95% confidence interval)

Figure 3 Pooled prevalence of iIFG in women (ADA threshold)

## iIFG prevalence in men (ADA threshold)

Method: Stuart-Ord (inverse double arcsine square root)

Non-combinability of studies

Cochran Q = 2,446.037587  (df = 6)  P < 0.0001

Moment-based estimate of between studies variance = 0.162683

I_2_ (inconsistency) = 99.8% (95% CI = 99.7% to 99.8%)

Random effects (DerSimonian-Laird)

Pooled proportion = 0.244905 (95% CI = 0.129259 to 0.383317)

Bias indicators

Begg-Mazumdar: Kendall's 0.238095  P = 0.5619

Egger: bias = 2.235259 (95% CI = -36.95331 to 41.423828)  P = 0.8892

Harbord: bias = -5.969634 (92.5% CI = -38.931193 to 26.991925)  P = 0.7015

**Prevalence of iIFG in men (ADA threshold)**

Figure 4 Pooled prevalence of iIFG in men (ADA threshold)

## **Prevalence of IGT/IFG**

## IGT/IFG prevalence in women (ADA threshold)

Method: Stuart-Ord (inverse double arcsine square root)

Non-combinability of studies

Cochran Q = 67.508559  (df = 4)  P < 0.0001

Moment-based estimate of between studies variance = 0.010078

I_2_ (inconsistency) = 94.1% (95% CI = 89.7% to 96.1%)

Random effects (DerSimonian-Laird)

Pooled proportion = 0.068121 (95% CI = 0.0467 to 0.093232)

Bias indicators

Begg-Mazumdar: Kendall's -0.2  P = 0.4833

Egger: bias = -5.570413 (95% CI = -13.577393 to 2.436568)  P = 0.1137

Harbord: bias = -4.48642 (92.5% CI = -8.777795 to -0.195044)  P = 0.0677

**Prevalence of IGT/IFG in men (ADA threshold)**

Figure 5 Pooled prevalence of IGT/IFG in women

## IGT/IFG prevalence in men (ADA threshold)

 Non-combinability of studies

Cochran Q = 222.504651  (df = 5)  P < 0.0001

Moment-based estimate of between studies variance = 0.016229

I_2_ (inconsistency) = 97.8% (95% CI = 97% to 98.2%)

**Random effects (DerSimonian-Laird)**

Pooled proportion = 0.084589 (95% CI = 0.057583 to 0.11622)

Bias indicators

Begg-Mazumdar: Kendall's -0.066667  P = 0.7194

Egger: bias = 2.592228 (95% CI = -11.072639 to 16.257095)  P = 0.6263

Harbord: bias = 0.360948 (92.5% CI = -12.594168 to 13.316063)  P = 0.9501

**Prevalence of IGT/IFG in men (ADA threshold)**

Figure 6 Pooled prevalence of IGT/IFG in men

## Odds ratio of IGT/IFG in women compared to men

Non-combinability of studies

 Breslow-Day = 92.408261  (df = 4)  P < 0.0001

Cochran Q = 88.993781  (df = 4)  P < 0.0001

Moment-based estimate of between studies variance = 0.450612

I_2_ (inconsistency) = 95.5% (95% CI = 92.8% to 96.9%)

**Random effects (DerSimonian-Laird)**

 Pooled odds ratio = 0.850624 (95% CI = 0.460192 to 1.572303)

Chi^2^ (test odds ratio differs from 1) = 0.266424  (df = 1)  P = 0.6057

 Bias indicators

 Begg-Mazumdar: Kendall's -0.2  P = 0.4833

Egger: bias = -5.997561 (95% CI = -12.981084 to 0.985962)  P = 0.0718

Harbord-Egger: bias = -5.619863 (92.5% CI = -10.966606 to -0.273119)  P = 0.0669

0.2

0.5

1

2

5

**Odds ratio of IGT/IFG in women compared to men (ADA)**

Cobb

1.04 (0.58, 1.86)

Novoa

0.55 (0.29, 1.04)

Markus

0.61 (0.44, 0.84)

Tamayo ST

0.66 (0.49, 0.88)

Satman

1.81 (1.63, 2.01)

combined [random]

0.85 (0.46, 1.57)

**Higher in men Higher in women**

**odds ratio (95% confidence interval)**

Figure 7 Forest plot of odds ratios of IGT/IFG in women compared to men

# Subgroup analyses

|  | **iIGT**  **w** | **I2** | **iIGT**  **m** | **I2** | **OR iIGT** | **I2** | **iIFG**  **w** | **I2** | **iIFG**  **m** | **I2** | **OR iIFG** | **I2** | **IGT/IFG**  **w** | **I2** | **IGT/IFG**  **m** | **I2** | **OR IGT/IFG** | **I2** |
| --- | --- | --- | --- | --- | --- | --- | --- | --- | --- | --- | --- | --- | --- | --- | --- | --- | --- | --- |
|  | **prev**  **95% CI** | **%**  **95% CI** | **prev**  **95% CI** | **%**  **95% CI** | **OR**  **95% CI** | **%**  **95% CI** | **prev**  **95% CI** | **%**  **95% CI** | **prev**  **95% CI** | **%**  **95% CI** | **OR**  **95% CI** | **%**  **95% CI** | **prev**  **95% CI** | **%**  **95% CI** | **prev**  **95% CI** | **%**  **95% CI** | **OR**  **95% CI** | **%**  **95% CI** |
| **All studies** | **0.08**  0.07, 0.09 | 85%  68.6%, 91% | **0.05**  0.04, 0.07 | 93.4%  89.8%, 95.3% | **1.42**  1.23, 1.65 | 52.8%  0%,  78% |  | | | | | | | | | | | |
| **ADA threshold only**  **(Sparso removed)** | **0.08**  0.07, 0.09 | 77.7%  36.4%, 88.2% | **0.06**  0.04, 0.07 | 94.3%  91.3%, 95.9% | **1.37**  1.12, 1.68 | 60.6% 0% to 81.9%) | **0.15**  0.12, 0.18 | 93.6%  89.4%, 95.7% | **0.24**  0.13, 0.38 | 99.8%  99.7%,99.8% | **0.65**  0.44, 0.96 | 95.7%  93.6% to 96.9% | **0.07**  0.05, 0.09 | 94.1%  89.7%, 96.1% | **0.08**  0.06, 0.12 | 97.8%  97%, 98.2% | **0.85**  0.46, 1.57 | 95.5%  92.8%, 96.9% |
| **Satman removed (Turkish study)** | **0.07**  0.06, 0.08 | 60.8%  0%, 81.9% | **0.05**  0.04, 0.07 | 91.5%  85.5%, 94.3% | **1.35**  1.13, 1.60 | 39.2%  0% to 74.7% | **0.15**  0.11, 0.19 | 94.9%  91.5%, 96.5% | **0.26**  0.15, 0.39 | 99.4%  99.3%, 99.5% | **0.59**  0.41, 0.86 | 90.8%  81.1%,94.4% | **0.06**  0.04, 0.08 | 83.8%  44.2%, 92% | **0.09**  0.07, 0.12 | 88.6%  74%, 93.3% | **0.67**  0.55, 0.81 | 6.9%  0%, 70.1% |
| **Small studies (<1,000 participants) removed** | **0.08**  0.07, 0.09 | 87.1%  69%, 92.7% | **0.05**  0.04,  0.07 | 94.9%  92.1%, 96.4% | **1.55**  1.40, 1.71 | 17%  0%, 69.8% | **0.15**  0.12, 0.19 | 95.8%  92.9%, 97.2% | **0.27**  0.13, 0.44 | 99.8%  99.8%, 99.8% | **0.62**  0.39, 0.97 | 96.8%  94.9%, 97.8% | **0.08**  0.05, 0.11 | 95.1%  89.6%, 97.1% | **0.09**  0.06, 0.13 | 98.6%  98.2%,98.9% | **0.91**  0.40,  2.05 | 97.4%  95.6%, 98.2% |
| **Recent data 2005-2010** | **0.08**  0.07, 0.10 | 81.1%  0%,  92.1% | **0.05**  0.03, 0.06 | 96.1%  93.4%,97.4% | **1.63**  1.48, 1.79 | 0%  0%, 72.9% | **0.14**  0.10, 0.19 | 96.7%  94.1%, 97.9% | **0.28**  0.12, 0.49 | 99.9%  99.9%, 99.9% | **0.56**  0.30, 1.07 | 97.9%  96.6%, 98.5% | **0.08**  0.05, 0.11 | 95.1%  89.6%, 97.1% | **0.09**  0.06, 0.13 | 98.6%  98.2%, 98.9% | **0.91**  0.40, 2.05 | 97.4%  95.6%, 98.2% |

Table 4 Sensitivity analyses (iIGT = isolated impaired glucose tolerance iIFG = isolated impaired fasting glucose IGT/IFG = combined IGT and IFG m= men w= women prev= prevalence)

**References Excluded studies**

1. Chen, G. *et al.* Comparison of Insulin Resistance and beta-Cell Dysfunction Between the Young and the Elderly in Normal Glucose Tolerance and Prediabetes Population: A Prospective Study. *Hormone and Metabolic Research* **49**, 135–141 (2017).

2. Kato, K. *et al.* Elevated C-reactive Protein Levels Independently Predict the Development of Prediabetes Markers in Subjects with Normal Glucose Regulation. *Exp Clin Endocrinol Diabetes* **129**, 289–295 (2021).

3. Hutchinson, M. S. *et al.* Serum 25-Hydroxyvitamin D levels in subjects with reduced glucose tolerance and type 2 diabetes-The Tromso OGTT-Study. *International Journal for Vitamin and Nutrition Research* **81**, 317–327 (2011).

4. Hung, H. C. *et al.* Associations between GDF15 Levels and Pre-Diabetes in Non-Obese Subjects. *Journal of Investigative Medicine* **70**, 79–84 (2022).

5. Gylling, H. *et al.* Cholesterol metabolism in relation to glucose tolerance in a large finnish male population. *Atheroscler Suppl* **10**, (2009).

6. De Vegt F. *et al.* Elevated fasting and postload glucose concentrations: Risk factors for developing type-2 diabetes mellitus; results of the Hoorn study. *Ned Tijdschr Geneeskd* **147**, 1414–1418 (2003).

7. Kohlenberg, J. D. *et al.* Differential contribution of alpha and beta cell dysfunction to impaired fasting glucose and impaired glucose tolerance. *Diabetologia* **66**, 201–212 (2023).

8. Valdes S. *et al.* Prevalence of diabetes and impaired glucose regulation in Spain: Di@bet.es study. *Diabetologia* **54**, S140 (2011).

9. Valdés, S. *et al.* Population-based incidence of type 2 diabetes in northern Spain: The Asturias study. *Diabetes Care* **30**, 2258–2263 (2007).

10. Rathmann W. *et al.* Incidence of Type 2 diabetes in the elderly German population and the effect of clinical and lifestyle risk factors: KORA S4/F4 cohort study. *Diabetic Medicine* **26**, 1212–1219 (2009).

11. Johansen, N. B. *et al.* Effect of regression or progression in glucose tolerance status on aortic stiffness: The ADDITION-PRO study. *Diabetes* **62**, A369–A370 (2013).

12. Linder, K. *et al.* Allele summation of diabetes risk genes predicts impaired glucose tolerance in female and obese individuals. *PLoS One* **7**, e38224 (2012).

13. Naeem, M. *et al.* Association of hepatic steatosis derived from ultrasound and quantitative MRI with prediabetes in the general population. *Sci Rep* **11**, 13276 (2021).

14. Naeem, M. *et al.* Association between hepatic iron overload assessed by magnetic resonance imaging and glucose intolerance states in the general population. *Nutrition, Metabolism and Cardiovascular Diseases* **32**, 1470–1476 (2022).

15. Yip, W. C. Y. Y. , Sequeira, I. R. , Plank, L. D. , & Poppitt, S. D. ,. Prevalence of pre-diabetes across ethnicities: A review of impaired fasting glucose (IFG) and impaired glucose tolerance (IGT) for classification of dysglycaemia. *Nutrients* **9**, 1273 (2017).

16. Eleftheriadou, A. *et al.* The prevalence of cardiac autonomic neuropathy in prediabetes: a systematic review. *Diabetologia* **64**, 288–303 (2021).

17. Meisinger, C. *et al.* Retinol-binding protein 4 is associated with prediabetes in adults from the general population: The cooperative health research in the Region of Augsburg (KORA) F4 study. *Diabetes Care* **34**, 1648–1650 (2011).

18. Meisinger C. *et al.* Serum potassium is associated with prediabetes and newly diagnosed diabetes in hypertensive adults from the general population: The KORA F4-Study. *Diabetologia* **56**, 484–491 (2013).

19. De Simone G. *et al.* Metabolic phenotypes and cardiovascular abnormalities in prediabetes. *J Am Coll Cardiol* **55**, A58 (2010).

20. Lorenzo, C. *et al.* Impaired fasting glucose and impaired glucose tolerance have distinct lipoprotein and apolipoprotein changes: The insulin resistance atherosclerosis study. *Journal of Clinical Endocrinology and Metabolism* **98**, 1622–1630 (2013).

21. Nichols, G. A., Arondekar, B. & Herman, W. H. Medical care costs one year after identification of hyperglycemia below the threshold for diabetes. *Med Care* **46**, 287–292 (2008).

22. Ohshita, K. *et al.* Elevated White Blood Cell Count in Subjects with Impaired Glucose Tolerance. *Diabetes Care* **27**, 491–496 (2004).

23. Liu, J. *et al.* Reduced Circulating Endothelial Progenitor Cells and Downregulated GTCPH i Pathway Related to Endothelial Dysfunction in Premenopausal Women with Isolated Impaired Glucose Tolerance. *Cardiol Res Pract* **2020**, 1278465 (2020).

24. Hong, S. M. *et al.* Glycated albumin is a useful indicator for screening impending diabetes and predicting beta cell dysfunction in the pre-diabetic condition. *Diabetologia* **57**, S123 (2014).

25. Hong, S. M. *et al.* Glycated albumin is a useful indicator for predicting beta cell dysfunction and impending diabetes in prediabetic condition. *Diabetes Res Clin Pract* **106**, S89–S90 (2014).

26. Wang, X. *et al.* A study comparing the prevalence of urinary albumin excretion and microalbuminuria in pre-diabetes subjects. *Zhonghua Nei Ke Za Zhi* **43**, 170–173 (2004).

27. Wang, Z. *et al.* Immunoglobulin e and mast cell proteases are potential risk factors of impaired fasting glucose and impaired glucose tolerance in humans. *Ann Med* **45**, 220–229 (2013).

28. Sasaki, N., Maeda, R., Ozono, R., Nakano, Y. & Higashi, Y. Association of obesity with serum free fatty acid levels in individuals at different stages of prediabetes. *Clin Obes* **12**, e12496 (2022).

29. Marunaka, Y. *et al.* Possibility of Venous Serum Cl- Concentration ([Cl-]s) as a Marker for Human Metabolic Status: Correlation of [Cl-]s to Age, Fasting Blood Sugar (FBS), and Glycated Hemoglobin (HbA1c). *Int J Mol Sci* **22**, (2021).

30. Pankow, J. S. *et al.* Cardiometabolic risk in impaired fasting glucose and impaired glucose tolerance: The atherosclerosis risk in communities study. *Diabetes Care* **30**, 325–331 (2007).

31. Nichols, G. A., Arondekar, B., Herman, W. H., G.A., N. & B., A. Complications of dysglycemia and medical costs associated with nondiabetic hyperglycemia. *Am J Manag Care* **14**, 791–798 (2008).

32. Piche M.E. *et al.* Impaired fasting glucose vs. glucose intolerance in pre-menopausal women: Distinct metabolic entities and cardiovascular risk? *Diabetic Medicine* **21**, 730–737 (2004).

33. Festa, A., Lorenzo, C., Wagenknecht, L. E., Hanley, A. J. G. & Haffner, S. M. Lipoproteins measured by conventional methods vs. Nuclear Magnetic Resonance (NMR) spectroscopy and association with glucose tolerance status: The Insulin Resistance Atherosclerosis Study (IRAS). *Diabetes* **60**, A190–A191 (2011).

34. Kim, S. H. & Reaven, G. M. Isolated Impaired Fasting Glucose and Peripheral Insulin SensitivityNot a simple relationship. *Diabetes Care* **31**, 347–352 (2008).

35. Staacakova, A. *et al.* Changes in insulin sensitivity and insulin release in relation to glycemia and glucose tolerance in 6,414 finnish men. *Diabetes* **58**, 1212–1221 (2009).

36. Markus, M. R. P. *et al.* Prediabetes is associated with lower brain gray matter volume in the general population. The Study of Health in Pomerania (SHIP). *Nutrition, Metabolism and Cardiovascular Diseases* **27**, 1114–1122 (2017).

37. Kowall, B. *et al.* Incidence rates of type 2 diabetes in people with impaired fasting glucose (ADA vs. WHO Criteria) and impaired glucose tolerance: Results from an older population (KORA S4/F4/FF4 Study). *Diabetes Care* **42**, E18–E20 (2019).

38. Ziegler, D. *et al.* Increased prevalence of cardiac autonomic dysfunction at different degrees of glucose intolerance in the general population: the KORA S4 survey. *Diabetologia* **58**, 1118–1128 (2015).

39. Meisinger, C. *et al.* Uric acid is more strongly associated with impaired glucose regulation in women than in men from the general population: The KORA F4-study. *PLoS One* **7**, e37180 (2012).

40. Meisinger, C. *et al.* Prevalence of undiagnosed diabetes and impaired glucose regulation in 35-59-year-old individuals in Southern Germany: The KORA F4 study. *Diabetic Medicine* **27**, 360–362 (2010).

41. Aye, M. M. *et al.* Progression of Impaired Glucose Regulation (IGR) to type 2 diabetes. *Diabetic Medicine* **27**, 33 (2010).

42. Giráldez-García, C. *et al.* The heterogeneity of reversion to normoglycemia according to prediabetes type is not explained by lifestyle factors. *Scientific Reports 2021 11:1* **11**, 1–11 (2021).

43. Faerch, K. & Vaag, A. Metabolic inflexibility is a common feature of impaired fasting glycaemia and impaired glucose tolerance. *Acta Diabetol* **48**, 349–353 (2011).

44. Cobb, J. E., Eckhart, A., Adam, K.-P., Pappan, K. & Sinnott, M. Metabolomic characterization of impaired fasting glucose (IFG) and impaired glucose tolerance (IGT). *Diabetes* **63**, A358 (2014).

45. Engberg, S. *et al.* Differential relationship between physical activity and progression to diabetes by glucose tolerance status: The Inter99 Study. *Diabetologia* **53**, 70–78 (2010).

46. Schmid, V. *et al.* Liver fat content and insulin secretion failure are associated with the long-term success of a lifestyle intervention to prevent type 2 diabetes: Results of the TULIP study. *Diabetologia* **59**, S171 (2016).

47. Tura, A. *et al.* Profiles of glucose metabolism in different prediabetes phenotypes, classified by fasting glycemia, 2-hour OGTT, glycated hemoglobin, and 1-hour OGTT: An IMI DIRECT study. *Diabetes* **70**, 2092–2106 (2021).

48. Lidegaard, L. P. L. P. *et al.* Physical activity energy expenditure vs cardiorespiratory fitness level in impaired glucose metabolism. *Diabetologia* **58**, 2709–2717 (2015).

49. Larsen, M. P. *et al.* Glucagon-Like Peptide 1: A Predictor of Type 2 Diabetes? *J Diabetes Res* **2017**, 7583506 (2017).

50. Liu, Y. *et al.* Evidence From a Systematic Review and Meta-Analysis: Classical Impaired Glucose Tolerance Should Be Divided Into Subgroups of Isolated Impaired Glucose Tolerance and Impaired Glucose Tolerance Combined With Impaired Fasting Glucose, According to the Risk . *Front Endocrinol (Lausanne)* **13**, (2022).

51. Gerstein, H. C. *et al.* Annual incidence and relative risk of diabetes in people with various categories of dysglycemia: A systematic overview and meta-analysis of prospective studies. *Diabetes Research and Clinical Practice* vol. 78 305–312 Preprint at https://doi.org/10.1016/j.diabres.2007.05.004 (2007).

52. Simonen, P. *et al.* Cholesterol metabolism in relation to glucose tolerance in a finnish male population. *J Diabetes* **1**, A47 (2009).

53. Faerch, K. *et al.* Impaired fasting glycaemia vs impaired glucose tolerance: Similar impairment of pancreatic alpha and beta cell function but differential roles of incretin hormones and insulin action. *Diabetologia* **51**, 853–861 (2008).

54. Duran, I. D. *et al.* Irisin levels in the progression of diabetes in sedentary women. *Clin Biochem* **48**, 1268–1272 (2015).

55. Borel, A. L. *et al.* Visceral, subcutaneous abdominal adiposity and liver fat content distribution in normal glucose tolerance, impaired fasting glucose and/or impaired glucose tolerance. *Int J Obes* **39**, 495–501 (2015).

56. Sokup, A., Szymanski, M. & Goralczyk, K. Impaired fasting glucose as a marker of heterogeneity of gestational diabetes mellitus. A study of 1025 women living in the region of Kuyavia and Pomerania in Poland. *Endokrynol Pol* **60**, 348–352 (2009).

57. Faerch, K. *et al.* Natural history of insulin sensitivity and insulin secretion in the progression from normal glucose tolerance to impaired fasting glycemia and impaired glucose tolerance: The inter99 study. *Diabetes Care* **32**, 439–444 (2009).

58. Pitchika, A. *et al.* Hepatic steatosis and hepatic iron overload modify the association of iron markers with glucose metabolism disorders and metabolic syndrome. *Liver International* **41**, 1841–1852 (2021).

59. de Vegt, F. *et al.* Relation of impaired fasting and postload glucose with incident type 2 diabetes in a Dutch population: The Hoorn study. *J Am Med Assoc* **285**, 2109–2113 (2001).

60. Iriarte-Campo, V. *et al.* *Incidence of T2DM and the Role of Baseline Glycaemic Status as a Determinant in a Metropolitan Population in Northern Madrid (Spain) - ScienceDirect*. *Diabetes Research and Clinical Practice* vol. 209 111119 (Elsevier, 2024).
